# Medusas petrifying gaze: Severe, diffused and refractory calcinosis from a patient with ACA-negative CREST syndrome

**DOI:** 10.1515/rir-2025-0015

**Published:** 2025-07-01

**Authors:** Lili Xu, Jie Wu, Shu Liang, Yilin Lu, Yilu Qin, Chao Zhang, Miaomiao Ma, Wenqiang Fan

**Affiliations:** The Fourth Clinical College of Xinxiang Medical University, Xinxiang 453000, Henan Province, China; Xinxiang Central Hospital, The Fourth Clinical College of Xinxiang Medical University, Xinxiang 453000, Henan Province, China; Xinxiang Key Laboratory of Diagnosis of Autoimmune Disease and Precision Pharmacotherapy, Xinxiang 453000, Henan Province, China; Henan Province Engineering Technology Research Center for Cell Therapy of Rheumatic and Immune Diseases, Xinxiang 453000, Henan Province, China

**Figure 1:**
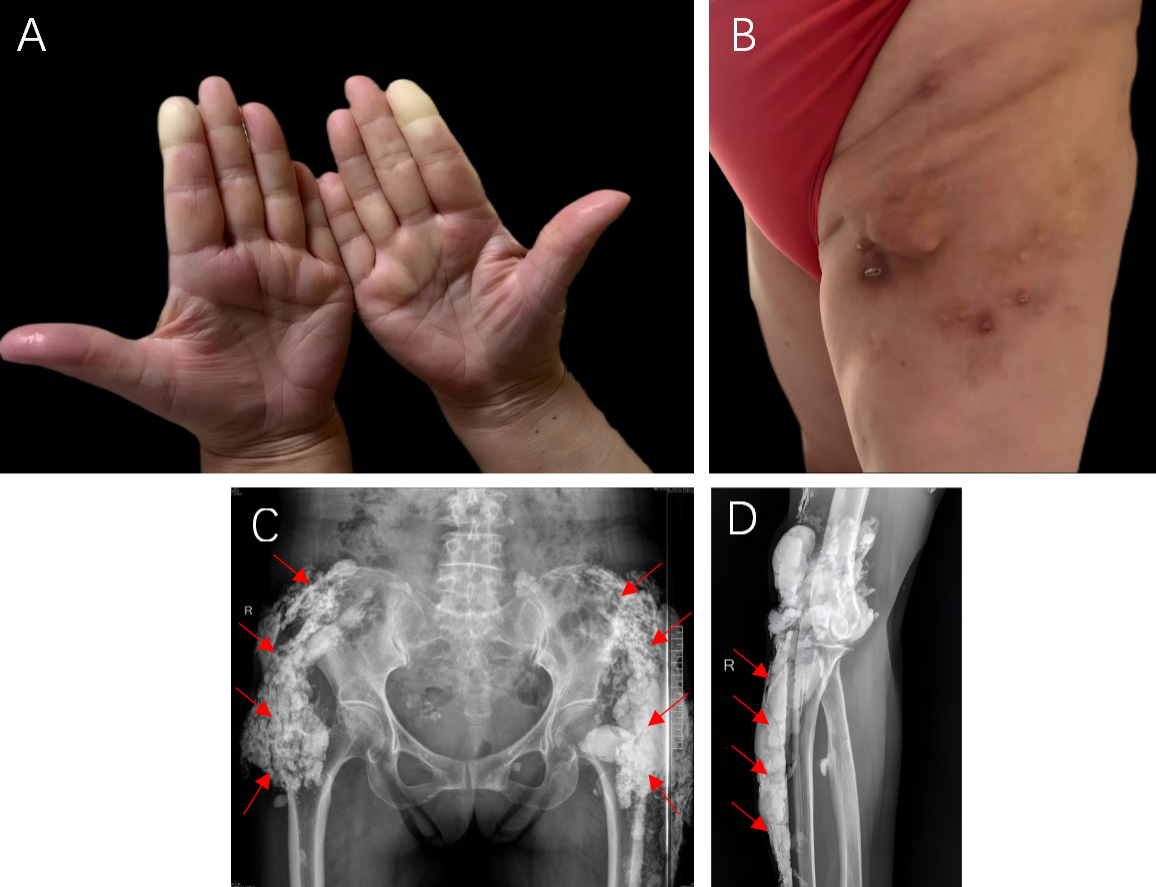
Clinical manifestations and imaging findings. A. Raynaud phenomenon. B. Multiple subcutaneous firm nodules on the extremities, which had ulcerated and released white, calcium-like material. C & D. X-rays of the upper extremities, pelvis, and femur showing multiple patchy calcifications in the pelvis, femur, and adjacent soft tissues.

A 62-year-old female with a decade-long history of Raynaud phenomenon (Figure 1A) developed xerostomia, esophageal dysmotility, and dysphagia, accompanied by severe disseminated subcutaneous calcifications predominantly affecting the hips and lower extremities (Figure 1B). Muscle strength remained normal (5/5) in all extremities. Laboratory examination revealed an elevated erythrocyte sedimentation rate (ESR) of 47 mm/h and a positive cytoplasmic-granular-pattern antinuclear antibody with a titer of 1∶80. Anti-centromere antibodies (ACA) were negative, and serum creatine kinase levels were within the normal range. Chest computed tomography (CT) showed interstitial lung disease (ILD). Radiographs of the right elbow, pelvis, and right femur demonstrated patchy soft tissue calcifications resembling gypsum (Figure 1C, 1D). Studies show that the sensitivity of ACA in diagnosing CREST syndrome is 65%, with a specificity of 99.9%.^[1]^ Consequently, a negative ACA alone cannot exclude the diagnosis, and clinical features along with other diagnostic findings remain crucial. Based on the available evidence, she was diagnosed with CREST syndrome complicated by ILD. The patient was treated with prednisone, cyclophosphamide, and hydroxychloroquine sulfate. Within two weeks, her Raynaud phenomenon and xerostomia improved, with ESR returning to normal. Three months later, the subcutaneous nodules had shrunk.

Calcinosis cutis refers to the deposition of insoluble calcium salts in the skin and subcutaneous tissues, typically in the form of dystrophic calcification and consisting primarily of hydroxyapatite crystals that resemble bone.^[2]^ The pathogenesis is poorly understood but vascular ischemia and repeated microtraumas are thought to be the key factors driving its development. It is commonly observed in conditions such as systemic sclerosis (SSc), systemic lupus erythematosus, and dermatomyositis. Calcinosis represents a great burden for SSc patients due to skin ulceration, infection, fistulation, and consequent disability, which significantly impairs patients’ quality of life. The most frequently affected areas are the hands and wrists, where calcinosis can lead to pain, limited joint mobility, ulceration, and secondary infections.^[3]^ In this case, the patient exhibited extensive and severe subcutaneous calcinosis, reminiscent of Medusa’s petrifying gaze, where calcification progressively spread from the skin into deeper tissues, severely restricting joint mobility.
